# Respiratory phase shapes brain-heart dynamics: evidence from recurrence analysis of EEG band power and heart rate

**DOI:** 10.3389/fnsys.2026.1905962

**Published:** 2026-07-31

**Authors:** MariNieves Pardo-Rodriguez, Erik Bojorges-Valdez, Oscar Arias-Carrion, Oscar Yanez-Suarez, Jorge Gonzalez-Ordiano

**Affiliations:** 1 Engineering Studies for Innovation, Universidad Iberoamericana Ciudad de México, Mexico City, Mexico; 2 Division of Clinical Neuroscience, Instituto Nacional de Rehabilitación Luis Guillermo Ibarra Ibarra, Mexico City, Mexico; 3 Neuroimage Research Laboratory, Universidad Autónoma Metropolitana-Iztapalapa, Mexico City, Mexico; 4 Institute of Applied Research and Technology, Universidad Iberoamericana Ciudad de Mexico, Mexico City, Mexico

**Keywords:** brain heart interactions, controlled breathing, non-linear dynamics, recurrence quantification analysis, respiratory modulation

## Abstract

**Introduction:**

Respiration is increasingly recognized as a key modulator of brain-body interactions, influencing both neural and autonomic dynamics. While prior work has demonstrated respiratory-driven entrainment, it remains unclear whether distinct phases of the breathing cycle give rise to different dynamical regimes of cortical activity.

**Methods:**

In this study, we investigated respiratory-phase-dependent modulation of brain-heart interactions during controlled breathing, focusing on breath-hold periods following inhalation and exhalation. EEG band power time series and heart rate (HR) were analyzed in 15 healthy subjects. In addition to conventional spectral measures, recurrence quantification analysis was employed to characterize non-linear dynamics in cortical activity.

**Results:**

Results revealed clear respiratory-phase-dependent differences. Inhale-hold was associated with increased γ band power, higher recurrence rate, and elevated HR, whereas exhale-hold showed reduced γ activity, lower recurrence, and decreased HR. These findings are consistent with known mechanisms of respiratory sinus arrhythmia and suggest that the two conditions correspond to distinct autonomic states. From a dynamical systems perspective, increased recurrence during inhale-hold suggests more structured and constrained cortical dynamics, while reduced recurrence during exhale-hold reflects comparatively more variable activity.

**Discussion:**

Together, these findings suggest that respiratory phase modulates the organization of brain-body dynamics, potentially reflecting shifts between more stable and more flexible regimes of neural activity. This phase-dependent organization supports the view that respiration may act as a physiological mechanism for dynamically tuning brain-heart interactions, with implications for understanding the role of breathing in the modulation of neural dynamics.

## 1 Introduction

The human brain and cardiovascular system form a tightly coupled network whose interaction unfolds across multiple timescales. Recent frameworks describe these interactions in terms of brain-body states, emerging from the continuous integration of neural, autonomic, and cardiovascular processes (Valenza et al., 2025; Villringer et al., 2025). Within this context, respiration plays a central role as a rhythmic physiological process that not only supports metabolic demands but also modulates both neural and autonomic activity (Ramirez and Baertsch, 2018). Far from being a simple automatic behavior, breathing is generated by a complex neural network and interacts with widespread brain systems involved in cognition, emotion, and physiological regulation (Ashhad et al., 2022). A growing body of evidence shows that respiration influences neural dynamics across multiple spatial and temporal scales, coordinating oscillatory activity and shaping interactions across distributed brain regions (Goheen et al., 2023; Tort et al., 2025). These effects are accompanied by concurrent changes in physiological variables such as heart rate or blood pressure and seem to affect brain network dynamics, highlighting the integrative role of respiration within the broader brain-body axis (Convertino et al., 2011; Critchley and Garfinkel, 2015; Zaccaro et al., 2018).

The interaction between neural and cardiovascular systems is mediated by bidirectional pathways linking the central and autonomic nervous system, forming what is commonly referred to as the brain-heart axis (Valenza et al., 2025). Within this framework, heart rate variability (HRV) reflects the dynamic balance between sympathetic and parasympathetic activity and serves as an accessible marker of autonomic regulation. A key manifestation of this interaction is respiratory sinus arrhythmia (RSA), whereby heart rate increases during inhalation and decreases during exhalation due to respiratory-phase-dependent modulation of vagal tone (Bernston et al., 1993). This effect arises from the interplay between pulmonary afferents and arterial baroreceptors, which together shape cardiovascular responses through brainstem circuits (Chapleau, 2023; Noble and Hochman, 2019). These mechanisms underscore the close coupling between respiration and autonomic function, providing a physiological basis for respiration-driven modulation of brain activity (Smith et al., 2017).

Building on these physiological mechanisms, recent studies have explored how controlled breathing shapes brain-heart interactions by examining the coupling between neural oscillations and autonomic signals. Analyses of EEG band power time series (BPts) and HRV have revealed bidirectional relationships, with specific components aligning with the breathing rhythm and mediating communication between cortical and autonomic systems (Pardo-Rodriguez et al., 2025). These findings suggest that respiration can act as a driving rhythm that entrains both neural and physiological dynamics, supporting coordinated regulation of bodily states (Klimesch, 2018; Lakatos et al., 2019). Notably, higher-frequency oscillations, particularly in the γ band, have been implicated as key contributors to this interaction, potentially reflecting fast integrative processes within the brain-heart axis (Pardo-Rodriguez et al., 2021, 2025; Umeno et al., 2003).

Despite these advances, it remains unclear whether different phases of the respiratory cycle give rise to distinct patterns of neural and autonomic dynamics. Most studies have focused on global measures of coupling or entrainment (Herrero et al., 2018; Tort et al., 2018), without explicitly addressing how phase-specific respiratory processes may differentially shape cortical activity. Given that inhalation and exhalation are associated with distinct autonomic states, reflecting shifts in sympathovagal balance, it is plausible that these phases correspond to different dynamical regimes of brain-body activity. From a dynamical systems perspective, such differences may reflect shifts in the organization of neural activity within a structured state space, where varying levels of stability and variability characterize distinct functional configurations (Deco et al., 2017; Poil et al., 2012; Stam, 2005; Tognoli and Kelso, 2014; Villringer et al., 2025).

In the present study, we investigate this possibility by analyzing EEG band power dynamics and heart rate during controlled breathing tasks, with a specific focus on breath-hold (voluntary apnea) periods following inhalation and exhalation. Using both conventional spectral measures and recurrence quantification analysis (Marwan et al., 2007; Webber and Zbilut, 1994), we assess whether these conditions are associated with distinct neural and autonomic signatures. We hypothesize that, although both conditions exhibit respiratory-driven entrainment (Pardo-Rodriguez et al., 2025), inhale-hold and exhale-hold will differ in their underlying dynamical organization, reflecting respiratory-phase-dependent shifts in the stability and flexibility of cortical dynamics.

## 2 Materials and methods

### 2.1 Data

This work builds upon the analysis of data described in Pardo-Rodriguez et al. (2025), where full details of the experiment and participant pool can be found. Briefly, data were collected from 15 healthy subjects (eight male, aged 24 ± 3 years), all of whom provided informed consent. The experimental protocol adhered to the Declaration of Helsinki and received approval from the Universidad Iberoamericana Ethics Committee under record number: Folio 221. EEG signals were recorded from 16 channels placed according to the 10–20 system, and ECG signals were recorded using chest electrodes placed below the left and right clavicles. The current study extends this initial work by further investigating the dynamic coupling between EEG band power and heart rate during conscious breathing, with a particular focus on breath-hold periods and new analyses of recurrence dynamics. Although the same experimental dataset and pre-processing pipeline were used, all breath-hold segmentation, statistical analyses, recurrence quantification analyses, and respiratory-phase-specific comparisons presented here are novel.

### 2.2 Experimental protocol

The original experimental protocol involved three conditions: spontaneous breathing (rest), followed by two controlled breathing tasks (CBT1 and CBT2), which were always performed in this order. In CBT1, subjects performed breathing cycles with 4 s of inhalation, 7 s of breath-holding, and 8 s of exhalation. CBT2 consisted of 5 s of inhalation, 8 s of exhalation, and 5 s of breath-holding. Both CBTs were performed for 10 min, yielding 32 cycles of CBT1 and 33 cycles of CBT2. For the current paper, the focus is on the dynamics observed during the breath-hold periods of each CBT where condition-specific differences are expected to emerge. Specifically, the analysis of band power time series (BPts) and recurrence rate (RR) were conducted to explore differences in neural dynamics between the inhale-hold and exhale-hold conditions.

The breathing protocols were selected to produce slow respiratory cycles with a total duration of approximately 20 s (~0.05 Hz), corresponding to a lower harmonic of the well-studied 0.1 Hz resonance frequency associated with enhanced parasympathetic activity and autonomic regulation (Zaccaro et al., 2018). The 4–7–8 protocol is a widely used pranayama-inspired breathing technique that has been associated with effects on HRV, blood pressure, and stress reduction (Vierra et al., 2022). The 5–8–5 protocol was designed to preserve a comparable overall cycle duration while shifting the breath-hold to the expiratory phase, thereby enabling direct comparison of respiratory-phase-dependent neural and autonomic dynamics under otherwise similar slow-breathing conditions, consistent with established respiratory training paradigms (Bhargava et al., 1988).

### 2.3 Signal processing

EEG and ECG data were processed using the same pipeline as described in Pardo-Rodriguez et al. (2025). EEG signals were band-pass filtered between 0.01 and 100 Hz, and relative BPts were computed for the five classical frequency bands: δ (1–4 Hz), θ (4–8 Hz), α (8–12 Hz), β (12–30 Hz), and γ (30–100 Hz). From the ECG data, heart rate variability (HRV) was computed from the time differences between consecutive R-peaks and then interpolated to a sampling rate of 10 samples per second (sps). The BPts signals were also resampled at 10 sps.

### 2.4 Analyses

#### 2.4.1 Band power time series (BPts) breath-hold analysis

**Figure 1:**
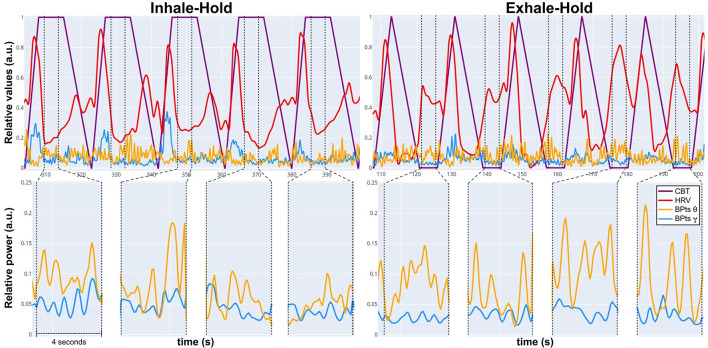
**Example of signals obtained and analyzed across conditions**. Each panel represents data from a single subject. The left panels correspond to the inhale-hold condition, and the right panels to the exhale-hold condition. The top-row panels display, in purple, the controlled breathing task (CBT); in red, the heart rate variability (HRV) signal normalized between 0 and 1; and in light orange and blue, the band power time series (BPts) for the θ and γ bands, respectively, from channel *Cz*. Vertical dashed lines indicate the 4-s windows centered on each cycle's breath-hold period. The second row zooms in on select windows, showing the corresponding BPts_θ_ and BPts_γ_ activity extracted for analysis.

To examine differences in band power dynamics between inhale-hold and exhale-hold conditions, 4-s breath-hold windows were extracted from each breathing cycle across all conditions (Figure 1). The window duration was chosen to maximize comparability between protocols while ensuring that all analyzed segments remained entirely within the breath-hold period despite the different breath-hold durations (7 s in CBT1 and 5 s in CBT2), leaving at least 0.5 s from the adjacent inhalation or exhalation phases. The windows were centered on the breath-hold period of each cycle, and BPts activity was extracted for each subject, frequency band, channel, and condition. This approach enables a direct comparison between conditions by restricting the analysis to equal-length segments of the breath-hold period.

Statistical analyses were conducted at two levels. At the single-subject level, inhale-hold and exhale-hold epochs yielded different numbers of analyzable windows and therefore had no natural one-to-one correspondence between cycles. Consequently, cycle-level comparisons were performed using Mann–Whitney *U*-tests. This initial analysis focused on midline channels (*Cz, Pz*, and *Oz*), selected because they provide a representative anterior-posterior sampling of the midline scalp distribution and have been previously implicated in respiratory-related cortical modulation. These channels were used to reduce dimensionality while preserving the main spatial pattern of the observed effects. For each subject, statistical comparisons were conducted across five frequency bands and three EEG channels (15 tests), and the resulting p-values were adjusted for multiple comparisons using the Benjamini–Hochberg False Discovery Rate (FDR) procedure. Adjusted p-values below 0.01 were considered statistically significant. Based on the single-subject analysis, subsequent analyses were restricted to the θ and γ bands, which exhibited the most pronounced and consistent condition-dependent modulation across subjects.

At the group level, the mean BPts value was computed across all extracted cycles for each subject, band, channel, and condition, yielding one paired observation per subject and condition. Group-level comparisons were performed using paired *t*-tests when both distributions satisfied the Shapiro–Wilk normality test, and Wilcoxon signed-rank tests otherwise. Group-level results are hereafter reported together with mean differences, 95% confidence intervals estimated by bootstrap resampling (5,000 iterations), and appropriate effect sizes (Cohen's *d* for *t*-tests and rank-biserial correlation for Wilcoxon tests). Lastly, to account for multiple comparisons across scalp locations, FDR correction was applied across all channel-wise comparisons within each frequency band.

#### 2.4.2 Recurrence analysis

**Figure 2:**
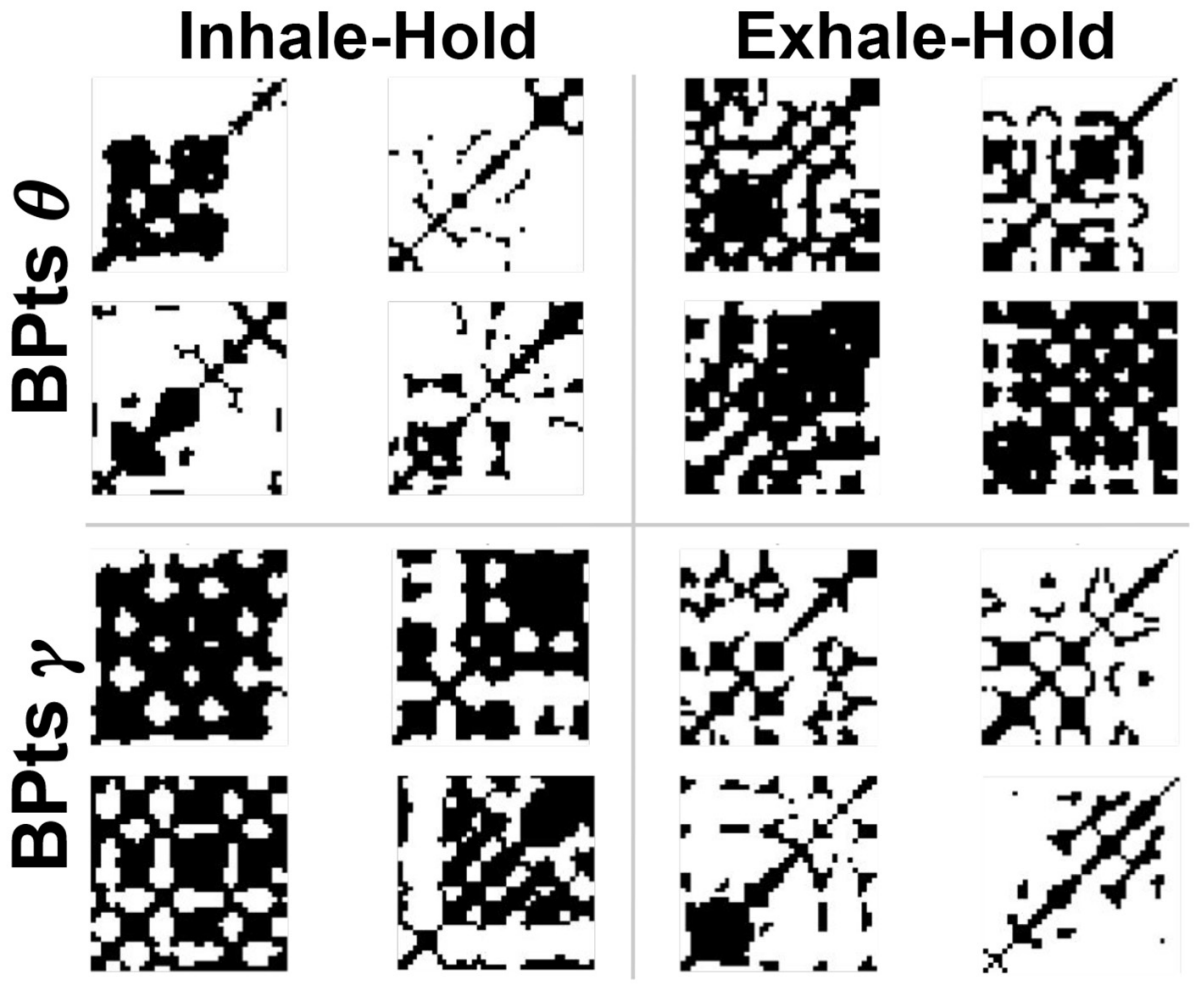
**Example of recurrence plots across conditions**. Recurrence plots from a single subject are shown. The top row corresponds to BPts_θ_, and the bottom row to BPts_γ_. Columns represent inhale-hold (left) and exhale-hold (right) conditions. Within each band and condition, four recurrence plots are displayed, each corresponding to a different breath-hold cycle (4-s window), yielding a total of 16 plots. Each recurrence plot was computed from multivariate signals constructed using BPts from channels *Cz, Pz*, and *Oz*. In this illustrative example, BPts_θ_ exhibits a higher recurrence rate during exhale-hold compared to inhale-hold, whereas the opposite pattern is observed for BPts_γ_.

To characterize non-linear dynamics in cortical activity during breath-hold conditions, recurrence quantification analysis (RQA) (Marwan et al., 2007; Webber and Zbilut, 1994) was performed on BPts from channels *Cz, Pz*, and *Oz* across subjects. Based on the previous analysis, this step focused on the θ and γ bands. For each trial and band, 4-s windows centered on the breath-hold period were extracted as described above (Figure 1) and standardized to remove amplitude bias between conditions. Multivariate recurrence plots were then constructed by combining *Cz, Pz*, and *Oz* signals using a three-dimensional embedding and the Manhattan distance metric. Figure 2 shows an example of recurrence plots from a single subject, illustrating four breath-hold cycles for each band and condition.

A critical step in the analysis was the selection of the recurrence threshold (ϵ). For each subject, ϵ was initially set to 1.5 standard deviations, ensuring robust recurrence structures while avoiding spurious matches. Additional values within a lower range were explored to assess the stability of recurrence measures. The resulting ϵ values consistently ranged between 1.2 and 1.5 across subjects for both γ and θ bands, with little variation in the pattern of results across this range, supporting the robustness of the findings. Importantly, the direction and relative magnitude of condition differences were preserved across this range.

From the recurrence plots, the recurrence rate (RR) was computed for each cycle. Statistical comparisons between inhale-hold and exhale-hold conditions were performed at the cycle level for each subject using independent-samples tests (Student's *t*-test when both distributions satisfied the Shapiro–Wilk normality test, and Mann–Whitney *U*-tests otherwise) across all available cycles (32 for inhale-hold and 33 for exhale-hold). For visualization purposes, RR values were subsequently averaged across cycles for each subject, band, and condition. Group-level differences between conditions were then assessed using paired *t*-tests separately for each frequency band. Mean differences, confidence intervals, and Cohen's *d* are reported.

This approach quantifies differences in the temporal organization and stability of BPts dynamics, capturing non-linear features that are not reflected in conventional spectral measures (Webber and Zbilut, 1994). Given that brain-heart interactions exhibit inherently non-linear and multifractal properties (Catrambone et al., 2021; Catrambone and Valenza, 2023), non-linear methods provide an effective complementary framework for characterizing the complex dynamics underlying brain-heart coupling.

#### 2.4.3 Heart rate (HR) analysis

The HRV signal was first converted to beats per minute (bpm) for each subject. The mean bpm value was then computed for each subject and condition. Group-level differences in HR between conditions were assessed using a paired *t*-test. Mean differences, confidence intervals, and Cohen's *d* are reported alongside *p*-values.

## 3 Results

### 3.1 HR analysis

**Figure 3:**
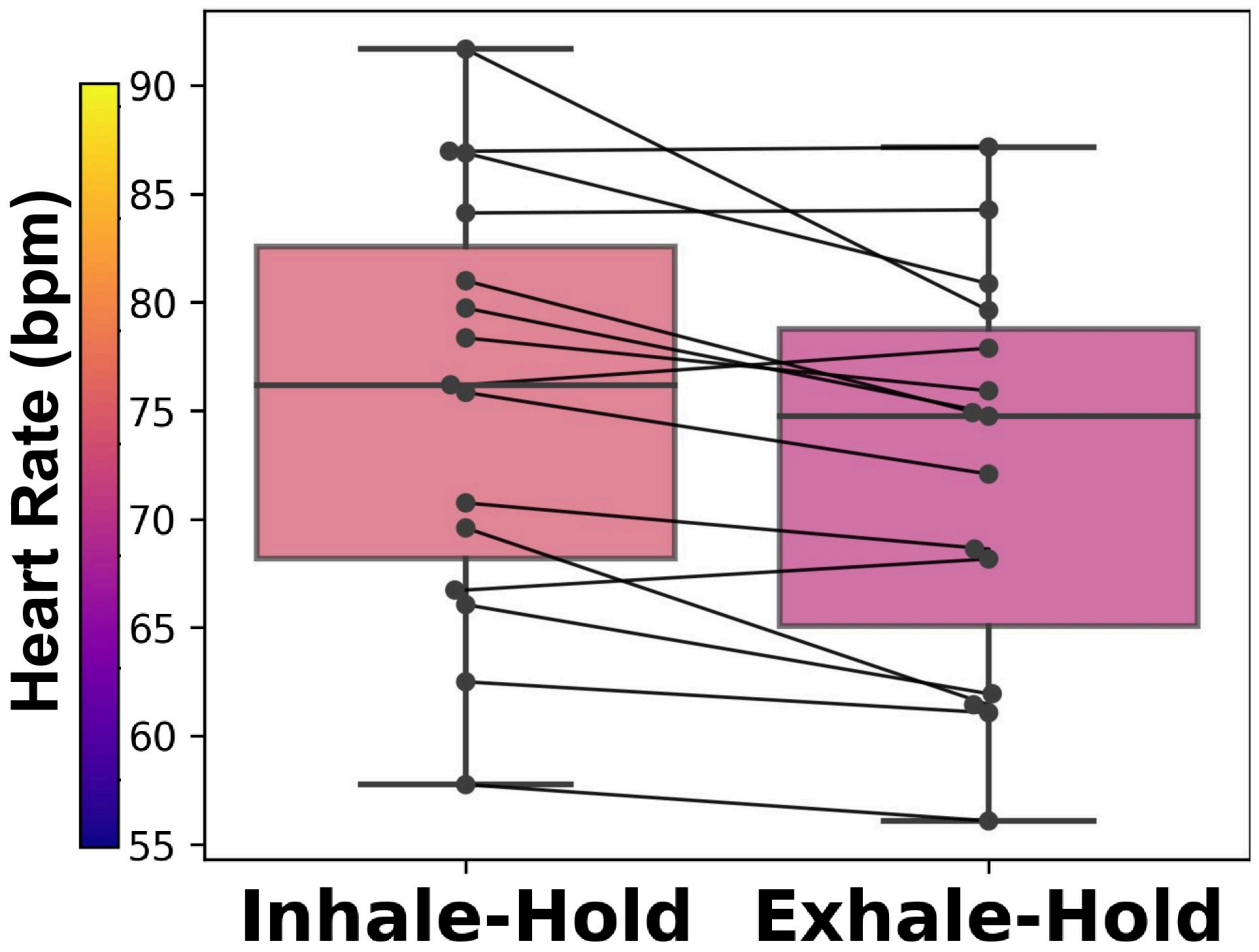
**Heart Rate Differences Between Conditions**. The panel displays paired boxplots of mean heart rate (HR) values for inhale-hold **(left)** and exhale-hold **(right)** conditions across subjects. Individual subjects are connected across conditions to highlight within-subject changes. Boxplots are colored according to the colorbar shown on the left, representing the mean HR across subjects for each condition in beats per minute (bpm). At the group level, HR is significantly higher for the inhale-hold condition compared to the exhale-hold condition (*p* < 0.01).

Figure 3 shows the mean heart rate (HR) across conditions. At the group level, HR was significantly higher during inhale-hold than during exhale-hold [*p* = 0.0045, mean difference = 3.293 bpm, 95% CI (1.527, 5.177), Cohen's d = 0.873]. This effect is consistent across subjects, as reflected by the predominance of higher HR values in the inhale-hold condition. The large effect size and confidence interval excluding zero further support the robustness of this difference. This result indicates that the two breathing conditions are associated with distinct cardiovascular states, likely reflecting differences in autonomic regulation and providing physiological context for the observed differences in BPts dynamics.

### 3.2 BPts breath-hold analysis

**Figure 4:**
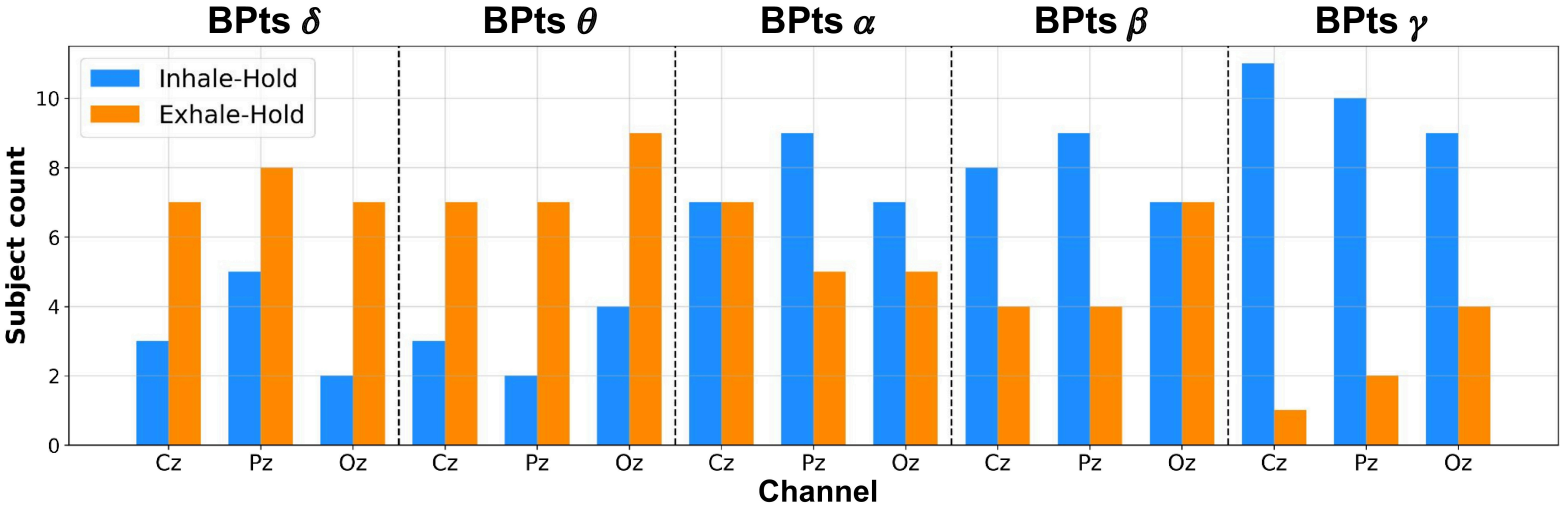
**Subject counts of significant Band Power time series (BPts) differences across conditions**. Bar plots show the number of subjects exhibiting significant differences in BPts between inhale-hold and exhale-hold conditions (FDR-corrected *p* < 0.01) across selected EEG channels (*Cz, Pz*, and *Oz*) and all frequency bands. Counts are assigned to the condition with greater power (inhale-hold, blue; exhale-hold, orange). Vertical separators indicate band divisions. The θ and γ bands exhibit the most pronounced differences between conditions across channels.

The BPts breath-hold analysis revealed that, for channels *Cz, Pz*, and *Oz*, between 9 and 14 out of 15 subjects exhibited significant differences between inhale-hold and exhale-hold conditions (FDR corrected *p* < 0.01). Across frequency bands, the most pronounced condition-specific effects were observed in the γ and θ bands. In the γ band, more than half of the subjects (>8) showed greater power during inhale-hold, with comparatively fewer subjects exhibiting higher power during exhale-hold (Figure 4). In contrast, the θ band displayed the opposite pattern, with more subjects showing increased power during exhale-hold. Based on these results, the γ and θ bands were selected for subsequent analyses, as they exhibited the clearest condition-dependent modulation of band power.

**Figure 5:**
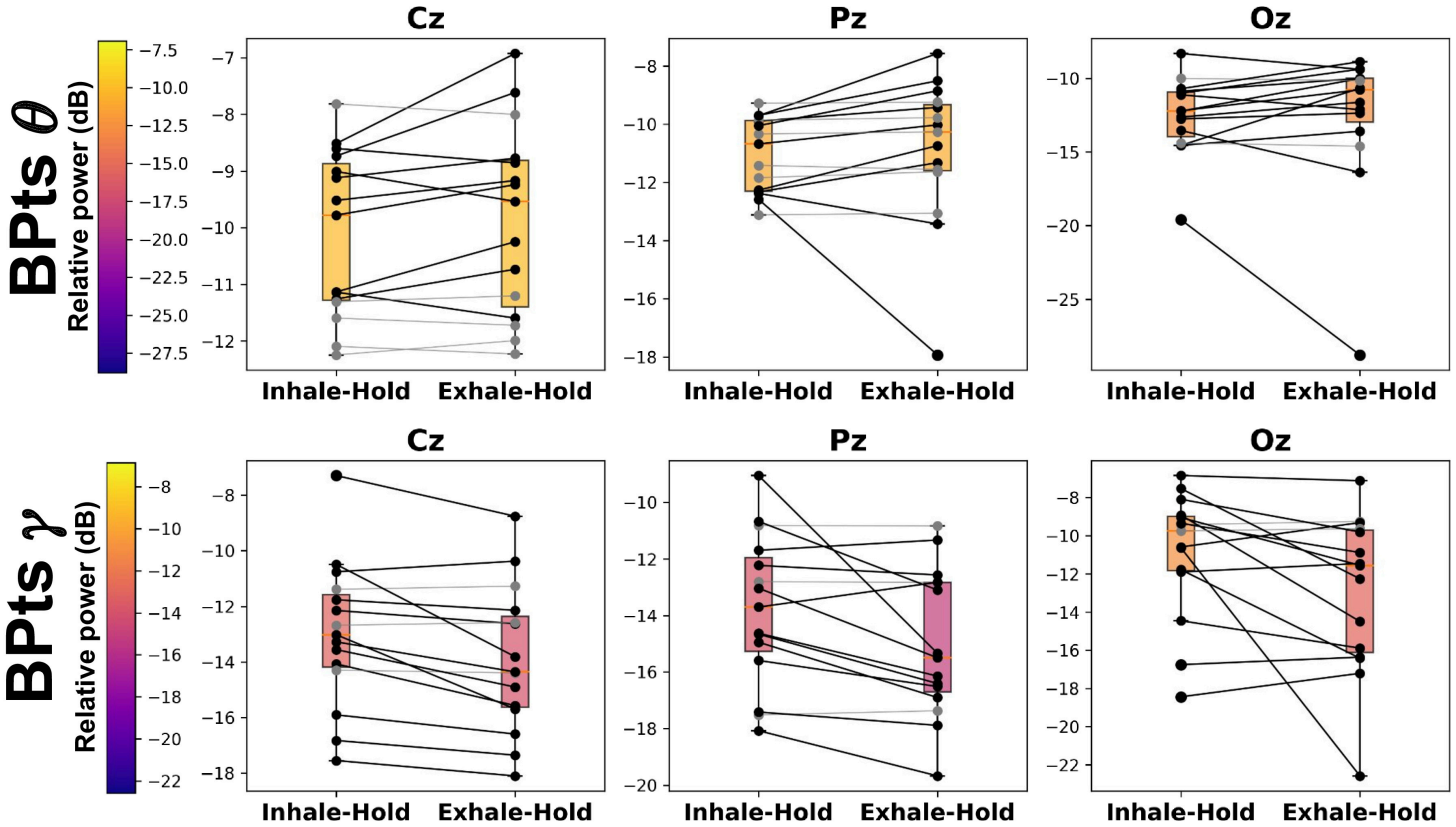
**Band Power time series (BPts) differences in** ***θ*** **and** ***γ*** **bands across conditions**. The top row shows BPts_θ_, and the bottom row shows BPts_γ_. Columns correspond to EEG channels *Cz, Pz*, and *Oz*. Each panel displays paired boxplots of mean BPts values for inhale-hold (left) and exhale-hold (right) conditions across subjects, with individual subjects connected by lines to highlight within-subject changes. Subjects exhibiting significant BPts changes (FDR-corrected *p* < 0.01) are shown in black, while non-significant cases are shown in gray. Boxplots are colored according to the colorbar displayed at the left of each row, representing the mean BPts value across subjects (in dB). Notably, a decrease in BPts_γ_ during exhale-hold is consistent across channels, while increases in BPts_θ_ during this condition are less evident, particularly at *Cz*.

Figure 5 presents the mean BPts values across cycles for each subject and condition, making the previously described patterns more explicit. In the γ band, a consistent decrease in BPts during exhale-hold is observed across all analyzed channels, reflected in lower group-level mean values compared to the inhale-hold condition. In contrast, the θ band shows a less consistent pattern, with increases during exhale-hold that are weaker and more variable across subjects, particularly at *Cz*.

**Figure 6:**
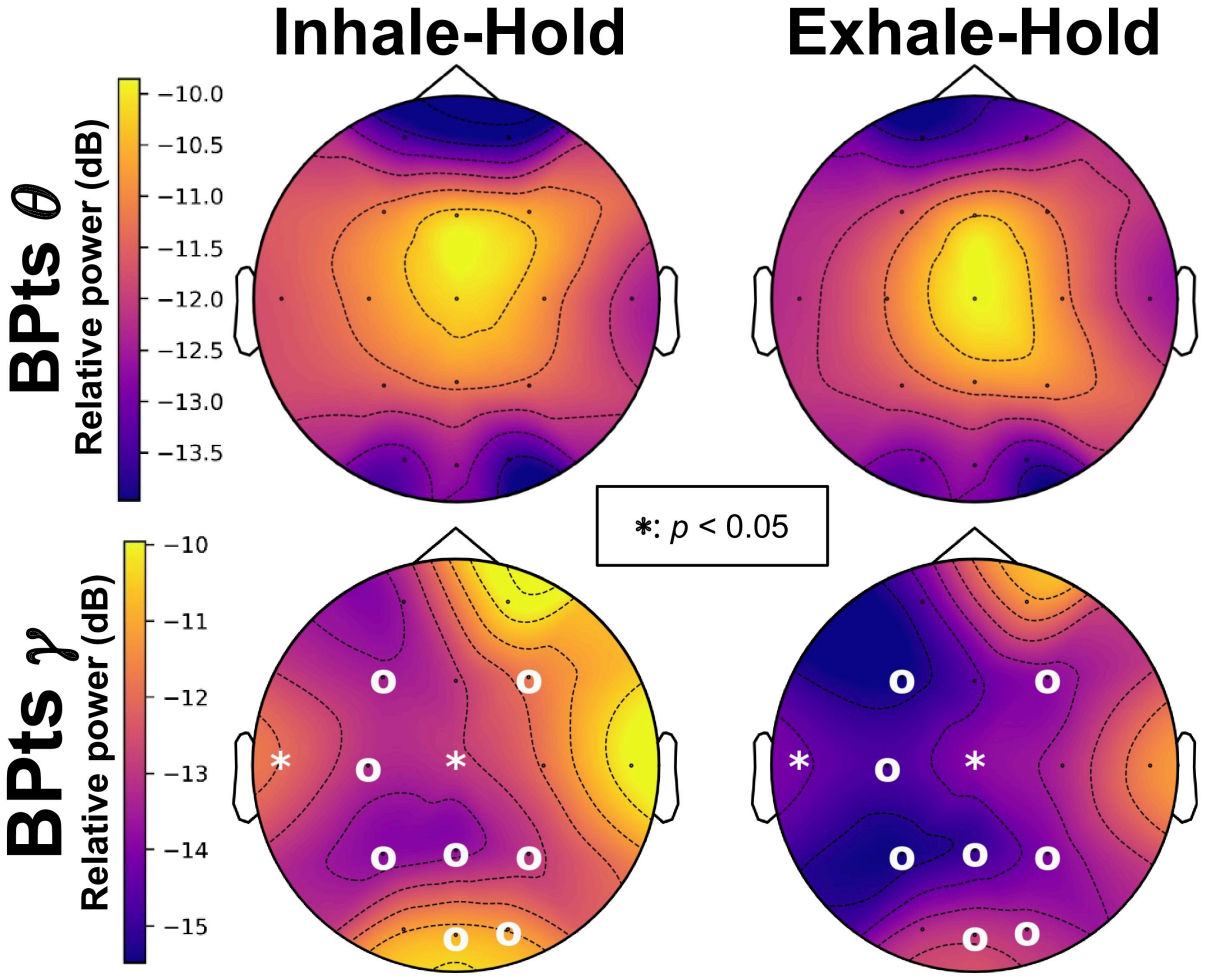
**Topographical distribution of mean BPts in** ***θ*** **and** ***γ*** **bands across conditions**. Scalp topographies show the mean BPts values across subjects for each EEG channel. The top row corresponds to BPts_θ_, and the bottom row to BPts_γ_. Columns represent inhale-hold **(left)** and exhale-hold **(right)** conditions. Group-level significant differences between conditions at each channel are indicated by white markers, denoting FDR-corrected significance thresholds (**o**: *p* < 0.1, *: *p* < 0.05). Significant effects were observed exclusively in the BPts_γ_ band, indicating that condition-related differences at the group level are driven primarily by γ activity.

These effects are further illustrated at the group level in Figure 6, which shows the spatial distribution of BPts values across all channels for each condition. In the γ band, after FDR correction, significant group-level differences remained at *Cz*, while several additional channels exhibited trends toward significance (*p* < 0.1). Although *T7* also remained significant after FDR correction, this isolated lateral effect is interpreted cautiously because of the unilateral reference configuration. In contrast, no significant group-level differences are observed in the θ band, suggesting that its condition-related modulation is less consistent across subjects.

Table 1 summarizes the group-level mean differences, 95% confidence intervals (CI), and effect sizes for the γ band. Except for *Fp1* and *Fp2*, the 95% CIs for the mean differences lay entirely above zero, consistent with greater γ band power during inhale-hold. The largest effect size was observed at *Cz*, followed by *P4* and *Pz*, with additional moderate effects over parietal and occipital regions.

**Table 1:** Mean BPts_γ_ differences between inhale-hold and exhale-hold conditions.

Channel	Mean (dB)	CI	Effect size	Channel	Mean (dB)	CI	Effect size
Fp1	1.125	[–0.710, 3.171]	0.282	Fp2	1.381	[–0.691, 3.608]	0.305
F3	2.025^*^	[0.246, 4.462]	0.481	F4	2.060^*^	[0.490, 4.189]	0.541
Fz	1.596	[0.295, 3.329]	0.503	Cz	0.902^**^	[0.433, 1.423]	0.871
C3	1.555^*^	[0.496, 3.048]	0.579	C4	1.439	[0.292, 3.070]	0.488
T7	2.262^**^	[0.882, 4.072]	0.696	T8	1.575	[0.026, 3.315]	0.453
P3	1.547^*^	[0.559, 2.829]	0.641	P4	1.013^*^	[0.349, 1.724]	0.717
Pz	1.227^*^	[0.469, 2.169]	0.701	Oz	2.046^*^	[0.521, 3.912]	0.584
O1	1.996	[0.239, 4.084]	0.502	O2	2.002^*^	[0.486, 3.811]	0.594

Group-level comparisons were performed across subjects for each EEG channel using either a paired two-tailed *t*-test or a Wilcoxon signed-rank test, depending on the normality of the data. FDR-corrected significant differences are indicated by ^*^ and ^**^, corresponding to *p* < 0.1 and *p* < 0.05, respectively. Positive values indicate greater BPts_γ_ during the inhale-hold condition. Mean differences, 95% confidence intervals (CI), and effect sizes are reported for each channel.

### 3.3 Recurrence analysis

**Figure 7:**
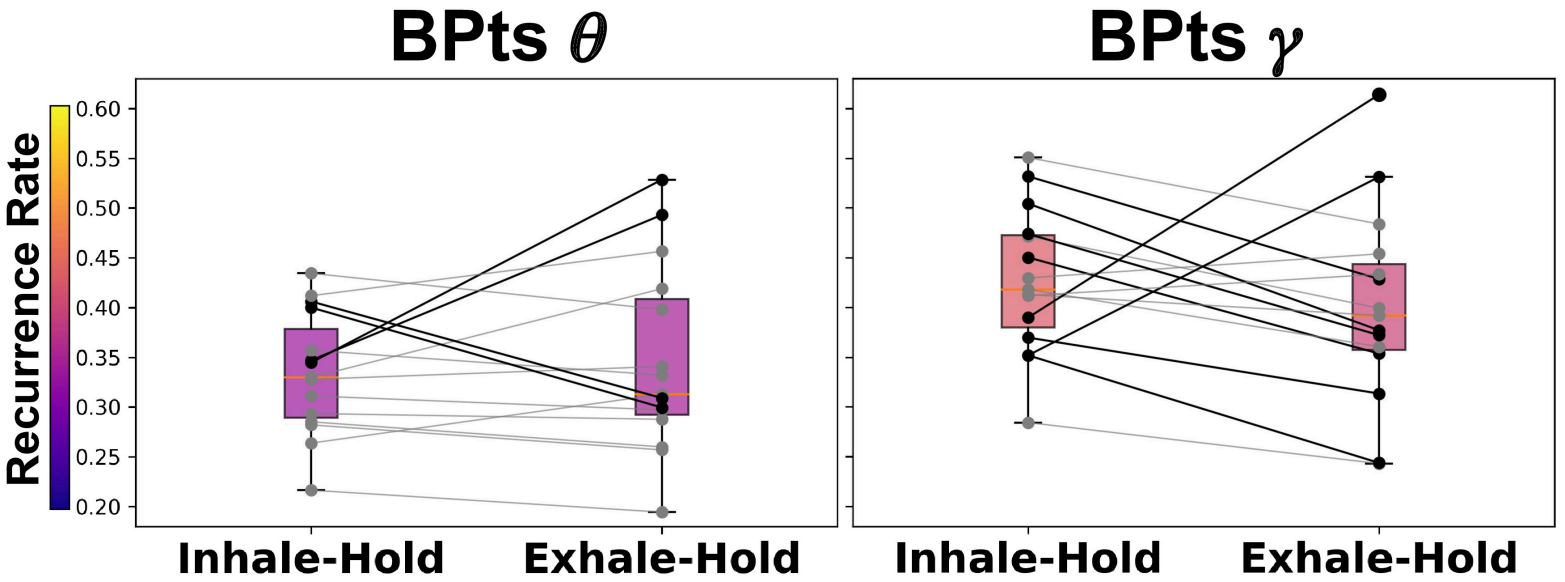
**Recurrence Rate Differences Across Conditions in** ***θ*** **and** ***γ*** **Bands**. The left panel shows results derived from BPts_θ_, and the right panel from BPts_γ_. Each panel displays paired boxplots of recurrence rate (RR) values for inhale-hold **(left)** and exhale-hold **(right)** conditions across subjects. Individual subjects are connected across conditions to highlight within-subject changes. Subjects exhibiting significant differences between conditions (*p* < 0.05) are shown in black, while non-significant cases are shown in gray. Boxplots are colored according to the colorbar displayed on the left, representing the mean RR across subjects. Notably, the γ band shows a clear difference in recurrence rate between conditions.

Figure 7 shows the recurrence rate (RR) across conditions for the θ and γ bands. In the γ band, most subjects exhibited higher RR values during inhale-hold compared to exhale-hold, with several subjects showing condition-related significant differences at the individual level. In contrast, the θ band displayed weaker and less consistent condition-related effects, with a slight tendency toward higher RR during exhale-hold.

At the group level, RR in the γ band showed a tendency toward higher values during inhale-hold (mean difference = 0.027, 95% CI [–0.028, 0.071], Cohen's *d* = 0.26), whereas the θ band showed a small tendency in the opposite direction (mean difference = –0.012, 95% CI [–0.053, 0.026], Cohen's *d* = −0.15). Neither comparison reached statistical significance, indicating that recurrence differences were relatively small and variable across subjects.

Additionally, RR values were overall higher in the γ band than in the θ band, indicating a greater degree of recurrent structure in faster oscillatory activity. These results suggest that respiratory-phase-related changes in recurrence dynamics were more apparent in the γ band, with increased recurrence predominantly associated with the inhale-hold condition, although the magnitude of these effects varied across individuals.

## 4 Discussion

The findings of the present study highlight the dynamic nature of brain-heart interactions, emphasizing the importance of respiratory-phase-dependent modulation on both neural and autonomic processes. Specifically, we observed distinct neural signatures during breath-hold periods following inhalation and exhalation, suggesting that these phases not only correspond to different autonomic states but also reflect underlying changes in the organization of brain dynamics. Together, these results extend previous findings of respiratory-driven entrainment by demonstrating that distinct phases of the breathing cycle are associated with different regimes of neural and autonomic dynamics. Rather than reflecting a uniform modulation, controlled breathing appears to partition brain-body activity into respiratory-phase-specific states, with inhale-hold favoring increased γ band power and more stable dynamics, and exhale-hold promoting comparatively reduced γ activity and more variable patterns.

It is worth noting, however, that although γ band activity exhibited greater respiratory-phase-dependent modulation across subjects, θ as well as α and β band power did not show consistent group-level differences between inhale- and exhale-hold conditions (Figure 6), suggesting that slower oscillations may be comparatively less sensitive to brief phase-specific respiratory perturbations in this context. Consistently, Figure 7 shows that θ-band dynamics exhibit only modest, variable trends across subjects, suggestive of a limited yet potentially contributory role in autonomic-cortical integration compared with the more robust effects observed in the γ band. Although analyses focused on bands exhibiting the most pronounced modulation, effects in other frequency bands cannot be fully excluded and may warrant further investigation.

The observed differences in HR between condition (Figure 3) are consistent with well-established mechanisms of RSA, in which cardiac activity is modulated by respiration through changes in vagal tone (Bernston et al., 1993). During inspiration, vagal activity is inhibited, leading to an increase in HR, whereas during expiration vagal activity is enhanced, resulting in a decrease in HR. In the present study, the inhale-hold condition can be interpreted as a sustained inspiratory state, and exhale-hold as a sustained expiratory state. Accordingly, the higher HR observed during inhale-hold and the lower HR during exhale-hold are consistent with a reduction and enhancement of vagal tone, respectively. These effects are likely further supported by baroreflex mechanisms, whereby changes in lung volume and intrathoracic pressure modulate blood pressure and baroreceptor activity, shaping vagal outflow through brainstem circuits (Chapleau, 2023; Convertino et al., 2011; Noble and Hochman, 2019). While the current study does not directly measure *CO*_2_, it is possible that the breath-hold conditions, by increasing *CO*_2_ levels, might engage chemoreflex responses. These could potentially contribute, in conjunction with vagal tone changes, to the observed differences in HR and neural activity in autonomic structures such as the brainstem and insular cortex (Critchley et al., 2015; Guyenet, 2014). Together, these mechanisms establish that the two conditions induce distinct autonomic states, providing a physiological basis for interpreting the concurrent differences observed in cortical dynamics.

Within this autonomic framework, the modulation of γ band activity suggests that cortical dynamics are sensitive to respiratory-phase-dependent changes in physiological state (Figures 4–6, and Table 1). The increased γ power and higher recurrence during inhale-hold point to a more coordinated and potentially more excitable mode of neural activity, whereas the reduced γ activity during exhale-hold indicates a comparatively less activated dynamical regime (Figures 2, 7). These differences, observed alongside changes in HR consistent with RSA, may reflect shifts in how autonomic state shapes cortical processing. These findings are consistent with the view of the brain-heart axis as a bidirectionally coupled system, in which autonomic signals continuously shape neural activity across multiple temporal scales (Smith et al., 2017; Valenza et al., 2025; Villringer et al., 2025). They also align with growing evidence that respiration acts as a global modulator of neural dynamics, influencing oscillatory activity and possibly cognitive emotional processes across widespread brain networks (Ashhad et al., 2022; Goheen et al., 2023; Tort et al., 2025).

From a dynamical systems perspective, these results suggest that respiratory phase modulates the location of brain activity within a structured state space, characterized by different levels of recurrence and variability. Recurrence measures provide a window into this organization by quantifying the tendency of a system to revisit similar states over time, thereby reflecting its stability and temporal structure (Marwan et al., 2007; Stam, 2005). Moreover, recurrence plots capture the deterministic structure of physiological systems even in the presence of non-stationary dynamics (Webber and Zbilut, 1994). In this context, the higher recurrence observed during inhale-hold may reflect a shift toward a more stable and constrained attractor-like region of the state space, whereas the reduced recurrence during exhale-hold can be interpreted as a transition toward more variable and and less predictable dynamics. Such phase-dependent transitions are consistent with theoretical frameworks proposing that healthy brain function operates near critical regimes, where an optimal balance between stability and flexibility supports efficient information processing and adaptability (Hesse and Gross, 2014; Poil et al., 2012).

Taken together, and building on this interpretation, respiration may act as a physiological mechanism that rhythmically perturbs and organizes brain-body dynamics, enabling transitions between distinct dynamical regimes across the breathing cycle (Goheen et al., 2023). Rather than maintaining a fixed operating point, the system appears to traverse a continuum of states shaped by ongoing autonomic and respiratory inputs. This perspective is compatible with frameworks of metastable brain dynamics, in which neural systems continuously transition between transiently coordinated states rather than remaining fixed in a single stable configuration (Deco et al., 2017; Tognoli and Kelso, 2014). This interpretation also aligns with recent perspectives on brain-body coupling, in which physiological signals such as HR and respiration continuously interact with neural activity to define transient functional states that underlie cognition and emotion (Villringer et al., 2025). In this framework, the differences observed between inhale-hold and exhale-hold reflect phase-specific configurations within a broader dynamical system.

Importantly, these findings extend prior work on respiratory-driven neural entrainment. Previous studies have shown that controlled breathing induces frequency-specific coupling between cortical activity and autonomic signals, with components of EEG band power aligning with the breathing rhythm and mediating bidirectional communication between brain and heart (Pardo-Rodriguez et al., 2025). The present results demonstrate that this coupling is not only frequency-specific but also respiratory-phase-dependent, affecting both the magnitude and the dynamical structure of cortical activity. The prominent involvement of the γ band further supports its role as a fast integrative mechanism within the brain-heart axis, potentially mediating rapid interactions between neural and autonomic processes (Pardo-Rodriguez et al., 2021, 2025; Umeno et al., 2003).

Consistent with this interpretation, brain-heart interactions have been shown to exhibit non-linear and multifractal dynamics that cannot be fully captured by linear measures alone (Catrambone et al., 2021). By employing recurrence quantification analysis, our findings suggest that non-linear dynamics may play a crucial role in the respiratory-phase-dependent regulation of brain and cardiovascular activity. This aligns with evidence that functional brain-heart interaction is dynamically reconfigured under different physiological and cognitive conditions, with distinct effects on vagal and sympathovagal dynamics (Catrambone and Valenza, 2023). In this study, the observed modulation of cortical dynamics and autonomic activity during inhale-hold and exhale-hold suggests a similar complexity, where different phases of the respiratory cycle lead to distinct functional states.

By demonstrating that inhale-hold and exhale-hold conditions are associated with distinct neural and autonomic configurations, the present study highlights the importance of considering respiratory phase when investigating brain-body interactions. This phase-dependent organization suggests that respiration may act as a physiological mechanism for dynamically tuning the balance between stability and flexibility in brain activity.

While these findings support a respiratory-phase-dependent organization of brain–heart dynamics, the controlled-breathing paradigm also involved sustained attention, temporal monitoring, and voluntary regulation of the breathing pattern. These cognitive and motor components have themselves been associated with modulation of cortical γ activity (Landau et al., 2007; Umeno et al., 2003; Zhang et al., 2025) and may therefore have contributed, together with respiratory-phase-dependent autonomic changes, to the observed effects. Thus, the present findings likely reflect the combined influence of respiratory phase and the cognitive demands inherent to the task. Future studies employing passive breathing manipulations, externally paced ventilation, or control tasks with comparable attentional demands will help dissociate these respective contributions.

Several limitations should be considered when interpreting these findings. First, the interpretation of recurrence measures as reflecting differences in stability or flexibility of neural dynamics remains indirect, as recurrence quantification captures temporal structure but does not provide a direct measure of the underlying neural mechanisms. Future work incorporating complementary non-linear metrics, such as entropy or complexity measures, may help further characterize these dynamical properties.

Second, since the study did not directly measure *CO*_2_ or *O*_2_ concentrations during the breathing tasks, we cannot determine how these concentrations varied over time or how their respective changes contributed to the observed effects, limiting the ability to assess the contribution of metabolic and chemoreflex-related mechanisms to the observed neural dynamics. Incorporating fNIRS measurements or direct respiratory gas monitoring in subsequent experiments could help clarify the physiological pathways underlying these interactions.

Furthermore, although the observed differences between inhale-hold and exhale-hold conditions are consistent with known autonomic mechanisms, the study does not include direct measures of sympathetic and parasympathetic activity beyond HRV, limiting the ability to fully disentangle their respective contributions. This limitation is partly related to the temporal constraints of HRV analysis, as the relatively slow dynamics of HRV make it less suitable for short windowed analyses aligned with breath-hold periods. Future studies incorporating additional autonomic measures such as blood pressure, electrodermal activity, or direct vagal indices with higher temporal resolution, may help clarify these mechanisms and provide a more comprehensive view of brain-body regulation.

The experimental design also introduces an important limitation. The two controlled breathing tasks were always performed in the same order, with CBT1 preceding CBT2, and task order was not counterbalanced across participants. Therefore, the observed differences between inhale-hold and exhale-hold conditions may partly reflect task-specific or order-related influences in addition to respiratory-phase-dependent effects. Although the average resting interval between tasks (4.29 ± 2.13 min) was intended to allow cardiovascular activity to return toward baseline and to minimize potential carry-over effects, future studies should counterbalance task order or incorporate both respiratory phases within a single experimental paradigm to more clearly isolate respiratory-phase effects from protocol-related influences.

Finally, although the sample size was comparable to previous exploratory psychophysiological studies investigating brain–body interactions (Critchley et al., 2000, 2015; De la Cruz-Armienta et al., 2017; Faes et al., 2017; Pfurtscheller et al., 2019; Valenza et al., 2016), the relatively small cohort limits the generalizability of the findings and motivates validation in larger samples. The effect sizes and confidence intervals reported here provide quantitative estimates that can inform future prospective power analyses and guide the design of larger studies aimed at characterizing respiratory-phase-dependent modulation of brain–heart dynamics. Furthermore, although the γ band findings were consistent across participants and analyses, future studies examining narrower γ sub-bands (e.g., low-γ) may further assess the specificity and robustness of these respiratory-phase effects.

## 5 Conclusion

In conclusion, our results support the view that the brain-heart axis operates as a non-linear, state-dependent system shaped by respiratory dynamics. By integrating physiological, neural, and dynamical perspectives, this work provides new insight into how controlled breathing modulates brain-body interactions. In particular, the observed phase-dependent differences suggest that respiration contributes to organizing brain activity across distinct dynamical regimes, associated with shifts in stability and variability. These findings also highlight the potential of non-linear analytical approaches to uncover mechanisms of physiological regulation and suggest that respiration-based interventions may offer a pathway for modulating brain-heart dynamics. Future research should determine whether the respiratory-phase-dependent dynamical states identified here generalize to spontaneous breathing and different clinical populations, and further investigate how these non-linear signatures relate to cognitive, affective, and autonomic processes.

## Data Availability

The raw data supporting the conclusions of this article will be made available by the authors, without undue reservation.
